# Multi- and Single-Joint Resistance Exercises Promote Similar Plantar Flexor Activation in Resistance Trained Men

**DOI:** 10.3390/ijerph17249487

**Published:** 2020-12-18

**Authors:** Paulo Gentil, Daniel Souza, Murillo Santana, Rafael Ribeiro Alves, Mário Hebling Campos, Ronei Pinto, Martim Bottaro

**Affiliations:** 1 College of Physical Education and Dance, Federal University of Goiás, Goiânia 74690-900, Brazil; daniel_souza86@hotmail.com (D.S.); murillohenrique12@hotmail.com (M.S.); alves.rafael.ribeiro@gmail.com (R.R.A.); mariohcampos@gmail.com (M.H.C.); 2 College of Physical Education, Federal University of Rio Grande do Sul, Porto Alegre 90040-060, Brazil; ronei.pinto@ufrgs.br; 3 College of Physical Education, University of Brasília, Brasília 70910-900, Brazil; martim@unb.br

**Keywords:** resistance training, muscle strength, rehabilitation

## Abstract

The present study aimed to compare soleus, lateral, and medial gastrocnemius muscles activation during leg press and calf raise exercises in trained men. The study involved 22 trained men (27.1 ± 3.6 years, 82.7 ± 6.6 kg, 177.5 ± 5.2 cm, 3.6 ± 1.4 experience years) who performed one set of each exercise using a 10-repetition maximum (10RM) load in a counterbalanced randomized order and separated by 10 min of rest. The electromyographic signal was measured for the three major plantar flexors: soleus, medial, and lateral gastrocnemius. A comparison between exercises showed that the mean adjusted by peak values during the leg press were 49.20% for the gastrocnemius lateralis, 51.31% for the gastrocnemius medialis, and 50.76% for the soleus. Values for calf raise were 50.70%, 52.19%, and 51.34% for the lateral, medial gastrocnemius, and soleus, respectively. There were no significant differences between exercises for any muscle (lateral gastrocnemius (*p* = 0.230), medial gastrocnemius (*p* = 0.668), and soleus (*p* = 0.535)). The present findings suggest that both leg press and calf raises can be used with the purpose to recruit triceps surae muscles. This bring the suggestion that one can chose between exercises based on personal preferences and practical aspects, without any negative impact on muscle activation.

## 1. Introduction

Resistance training (RT) is a popular mode of physical exercise often associated with voluntary muscle contraction against an external load, and its regular practice has been advised as an essential part of physical conditioning programs [1,2]. RT benefits are often associated with improvements in the musculoskeletal system such as increased muscle strength and hypertrophy, improved bone health [3,4], reduced low-back pain [5], and better functionality [6,7,8]. Such adaptations are influenced at some extend by the mechanical stimuli provided by repeated muscle activation in a process called mechanotransduction [9,10]. Muscle activation and subsequently mechanical stimuli might be affected by the manipulation and combination of RT variables such as load [11], rest between sets [12], and exercise selection [13,14]. 

Regarding exercise selection, RT can be performed with single (SJ) or multi-joint exercises (MJ), depending on the number of joints involved. For upper body muscles, it has been well established that SJ and MJ promote similar levels of muscle activation [15,16], as well as similar increases in muscle size and strength [17,18]. Moreover, other studies showed that, in general, the addition of SJ exercises to an RT program involving MJ exercises might not be necessary to bring optimal results in terms of muscle size and strength [19,20,21,22,23,24]. This brought the suggestion that one can chose between SJ and MJ based on personal preferences and practical aspects, without any negative impact on the results obtained from the intervention. Although less evidence is available for the lower body, studies about muscle activation [25,26], muscle strength [18], and hypertrophy [27] showed the same trends for thighs and hip muscles. However, information for calf muscles is scarce and controversial.

Bryanton et al. [28] reported near maximum efforts of the plantar flexors during the squat, when compared to the torque produced during a maximum isometric contraction. Interestingly, the relative muscle effort of the plantar flexors during the squat was similar to the ones of the hip and knee extensors, suggesting that the plantar flexors have an important involvement in the exercise performance. On the other hand, Escamilla et al. [29] reported that the activation of the plantar flexors was relatively low during different variations of the squat and leg press (9–17%), when normalized to a maximal isometric contraction. In addition to the controversial findings, these previous studies only analyzed MJ exercises and did not compare MJ and SJ under similar load conditions. Therefore, the current literature does not allow to conclude if plantar flexor muscle activation during a MJ exercise would be equivalent to the activation obtained during SJ exercises.

Some studies suggest that activation might be important for muscle adaptations [30], since mechanotransduction is likely to occur only in muscle fibers activated during exercise [31]. This suggests that high levels of muscle activation produced from repeated contractions can provide stimulation to the muscle, especially when combined with other factors, such as mechanical and metabolic stress, muscle swelling, etc. [31,32,33,34,35]. Therefore, considering that electromyographic (EMG) activity may reflect greater challenges to the musculoskeletal system, the investigation of plantar flexors muscle activity during MJ and SJ exercise using EMG analysis might contribute to close the gap regarding this topic.

Although plantar flexors are not commonly studied, their morphology and function might be important in clinical conditions such as chronic venous insufficiency and venous leg ulcers [36,37]. Moreover, strengthening the plantar flexors might have important applications in rehabilitation [38], to maintain orthostatic tolerance during exposure to microgravity [39], to improve balance [40,41], decrease the risk of falls [40], treat Achilles tendinopathy [42], and improve functionality [41]. Therefore, knowledge about how different exercises recruit these muscles might be of clinical and practical relevance. Based on the lack of information about plantar flexors’ activity during SJ and MJ exercises and the clinical and practical importance of the plantar flexors, the purpose of the present study was to compare soleus, lateral, and medial gastrocnemius muscles activation during leg press and calf raises in trained men. Our hypothesis is that calf raises would elicit superior activation of all muscles analyzed in comparison to leg press.

## 2. Materials and Methods

### 2.1. Experimental Approach

The study involved 22 trained men who visited the laboratory three times. The first two visits involved 10RM testing in the leg press and standing calf raise exercises and the third involved the performance of one set in each exercise using the 10RM load. Each visit was separated by 48–72 h and the volunteers were oriented to not perform any exercise or strenuous activity involving the studied muscles for 48 h before the first visit and between the subsequent visits. During the third visit, the exercises were performed in a counterbalanced randomized order and separated by 10 min of rest. Electromyographic signal was measured for the three major plantar flexors: soleus, medial, and lateral gastrocnemius.

### 2.2. Participants

Volunteers were recruited by social media and personal invitation among college students and the attendees of the Univesity’s RT facility. To be eligible to participate, the volunteers must have had at least one year of experience with RT, including the exercises tested. The performance of two RT sessions per week, involving 4 sets of exercise for the major muscle groups, was defined as minimum criteria to regular RT participation in accordance with the American College of Sports Medicine recommendations [1]. The volunteers were not allowed to participate if they had any clinical condition or medical problem that could be aggravated by the study protocol. After being informed about the study protocol, its risks and benefits, the participants signed and informed consent form in accordance with the Declaration of Helsinki. The study was approved by the relevant Ethics Committee (Protocol N°.56907716.5.0000.5083).

### 2.3. 10-Repetition Maximum (10RM) Testing

Participants performed 10RM tests on the leg press and calf raise in the first and second sessions in accordance with the National Strength and Conditioning Association (NSCA) recommendations [43]. Before the tests, the participants warmed up with 10 reps at a comfortable self-selected load and then rested for 5 min. Then, the initial load was defined based on the participants’ training history. If the volunteer could not perform 10 repetitions or performed more than 10 repetitions, the load was adjusted by 5–10% and another attempt was performed after 5 min of rest. No more than three attempts were necessary in any occasion. The tests were performed at a controlled velocity. A digital metronome was used, and the volunteers were oriented to perform each muscle action (concentric and eccentric phases) in two seconds, with no pause between contractions. The range of motion was controlled for each movement.

The leg press was performed in a 45-degree sled machine (Hammer Strength; Life Fitness, São Paulo, Brazil). The back pad was adjusted to provide a hip angle of 90 degree when the knees were fully extended. The movement went from full knee extension to 100 degrees of knee flexion. Calf raises was performed in standing position using a specific machine (Hammer Strength; Life Fitness, São Paulo, Brazil) with the knees fully extended during all time. Range of motion went from full ankle extension to full flexion. The movement was interrupted when the participants could not perform the movement with the defined range of motion or could not adhere to the proposed cadence [44].

### 2.4. Exercise Testing

**Figure 1:**
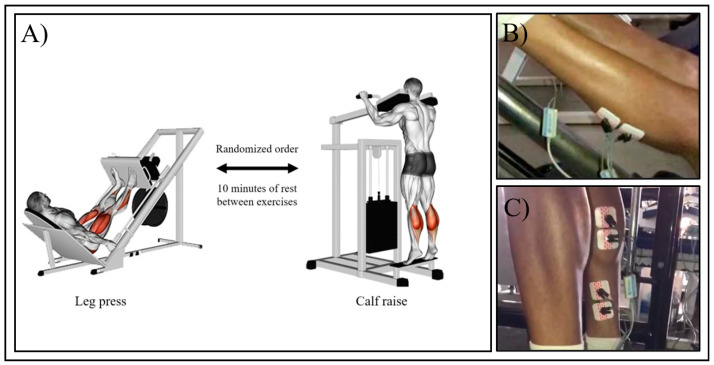
Illustration of exercise testing procedure (**A**), and electrode position during leg press (**B**) and calf raise (**C**).

The exercises were performed in the third visit using the load obtained in the 10RM testing and following the same procedures, with an especial attention to range of motion and movement velocity. We adopted a controlled movement velocity by controlling cadence, to standardize the time windows of the EMG signal and because a previous study showed that this could predict proximity to maximum effort [44]. The participants performed both exercises in the same day with the same electrode positioning. The exercises were performed in a randomized counterbalance order and were separated by 10 min of rest, as illustrated in Figure 1A. All tests and executions were recorded and reviewed to certify that they adhered to the procedures.

### 2.5. Electromyography

Electromyographic activity was recorded from the soleus, medial, and lateral gastrocnemius muscles. After skin preparation, including shaving and abrasion with alcohol to minimize impedance, pairs of electrodes were positioned in a bipolar configuration (distance of 20 mm between electrodes) along the direction of the muscle fibers according to the SENIAM recommendations (www.seniam.org) (Figure 1B,C). The reference electrode was placed on the knee patella. Electromyographic activity was measured using a system with 4 channels (Miotool400, 14-bit resolution, Miotec-Biomedical Equipment), and with a sampling frequency of 2000 Hz per channel. After measurement, electromyographic signals were filtered using the Butterworth filter with 20 Hz and 500 Hz cut-off frequencies for the lower and upper bandpass, respectively; and the adjusted by peak values were calculated while performing all repetitions. The means values were normalized using the maximum value obtained during the tests for each muscle.

### 2.6. Statistical Analysis

The normality of the data was confirmed by the Shapiro–Wilk test. Data are presented as means ± standard deviation. Paired T-tests were performed to compare mean EMG values between exercises. The effect size (ES) of the mean difference between exercise conditions was assessed by Cohen’s *d* effect size (mean exercise 1—mean exercise 2/SD_pooled_). An ES < 0.20 is considered trivial, small ≥ 0.20 and ≤ 0.60, moderate > 0.60 and ≤ 1.20, large > 1.20 and ≤ 2.00, and extremely large > 2.00 [45]. In addition, the agreement for the flexors plantar mean EMG values in leg press and calf raise was assessed by the simple linear regression and the Bland–Altman plot. An ES is considered as very weak if R^2^ < 0.04, weak if 0.04 ≤ R^2^ < 0.16, moderate if 0.16 ≤ R^2^ < 0.49, high if 0.49 ≤ R^2^ < 0.81 and very high if 0.81 ≤ R^2^ < 1.0 [46]. Statistical significance was defined as *p* < 0.05. Statistical analyses were performed using the Statistical Package for the Social Sciences 20.0 software (SPSS, Chicago, IL, USA).

## 3. Results

Participant characteristics are presented in Table 1. The load used for 10RM were (282.1 ± 32.7) in the leg press and (78.5 ± 7.8) in the calf raise. The estimated 1 repetition maximum [47] was 376 kg for the leg press and 105 kg for the calf raise.

**Figure 2:**
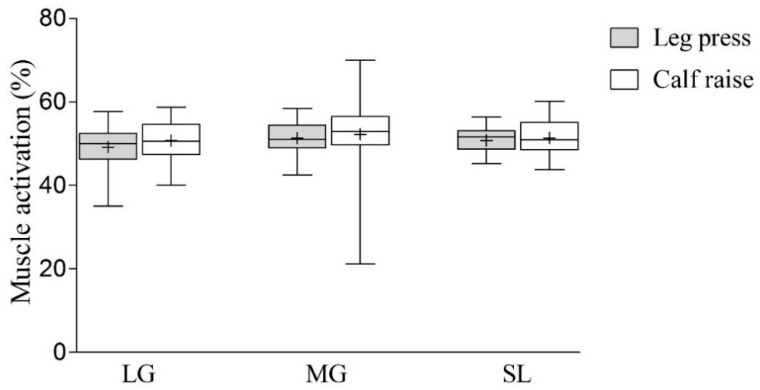
Comparison of muscle activation between leg press and calf raise. LG, lateral gastrocnemius; MG, medial gastrocnemius; SL, soleus. Values are presented as median (lines) with interquartile range (boxes) ± range (minimum and maximum) and + indicates mean.

Means values are illustrated in Figure 2. Comparison between groups showed that mean EMG values during the leg press were 49.2% for the lateral gastrocnemius, 51.31% for the medial gastrocnemius, and 50.76% for the soleus. Values for calf raises were 50.7%, 52.19%, and 51.34% for the lateral, medial gastrocnemius, and soleus, respectively. There were no differences between exercises between groups for any muscle and the ES of differences were trivial (gastrocnemius lateralis (*p* = 0.230, ES = 0.18), gastrocnemius medialis (*p* = 0.668, ES = 0.08), and soleus (*p* = 0.535, ES = 0.07)).

**Figure 3:**
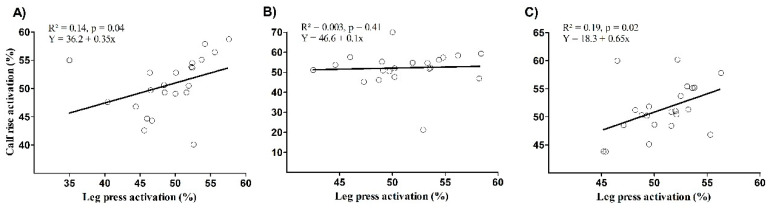
Correlation between calf raise and leg press mean EMG for the lateralis gastrocnemius (**A**), the medialis gastrocnemius (**B**), and the soleus (**C**).

**Figure 4:**
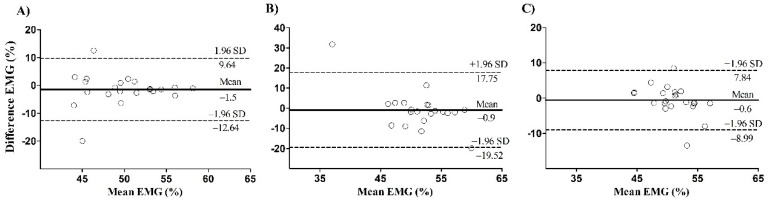
Bland–Altman plot of difference EMG for the lateralis gastrocnemius (**A**), the medialis gastrocnemius (**B**), and the soleus (**C**). The dotted line represents the limits of agreement upper and lower boundary. The continued line on the center of plot represents the systematic bias. The continued line on the Y axis represents the mean difference between leg press and calf raise mean EMG, and on the X axis represents the mean of leg press and calf raise mean EMG.

There is a significant correlation between the leg press and calf raise mean EMG for the lateral gastrocnemius (*p* = 0.04) and the soleus (*p* = 0.02), but not for the medialis gastrocnemius (*p* = 0.41) (Figure 3). The Bland–Altman plot showed systematic bias of −1.5 ± 5.7, −0.9 ± 9.5 and −0.6 ± 4.3 for the lateral, medial gastrocnemius, and soleus, respectively (Figure 4). The presence of proportional bias was verified for the medial gastrocnemius (*p* < 0.01), but not for the lateral gastrocnemius (*p* = 0.84) and soleus (*p* = 0.50).

## 4. Discussion

The purpose of the present study was to compare plantar flexors muscle activation during SJ and MJ exercises in trained men, here represented by calf raise and leg press exercises, respectively. Our main finding is that there was no difference for muscle activation for any muscle analyzed. This agrees with previous findings in upper and lower body muscles [15,16,25,26] and confirms the suggestion that plantar flexors have a great involvement in lower body MJ exercises [28,48]; however, the results were, to some extent, unexpected.

Plantar flexion is performed mainly by the three muscles that compose the triceps surae: soleus, medial, and lateral gastrocnemius. Soleus is originated in the tibia and fibula and inserted in the calcaneus, crossing only one joint. Considering that it acts mainly in the ankle and that both the leg press and calf raises exercises have a similar range of motion in that joint, the similar levels of muscle activation was not surprising. However, the medial and lateral gastrocnemius cross the knee and ankle joints, as they are originated in the femur and inserted in the calcaneus. Therefore, the gastrocnemius would have antagonistic actions during the leg press, as they flex the knee and extend the ankle, which is known as the Lombard Paradox [49]. Considering that the shortening in one joint is accompanied by the lengthening at the other, it was expected that the gastrocnemius activation would be decreased during the leg press. However, this was not the case. Considering that the calf muscles might act as dynamic knee stabilizers [50], one possible explanation is that the muscle contracted mainly isometrically, which can increase activation, as previously suggested [51].

To the best of our knowledge, this the first study to compare calf muscle activation between MJ and SJ exercises. The present results have many potentially important applications, since exercises oriented for the plantar flexors might be useful in chronic venous insufficiency and venous leg ulcers [36,37], rehabilitation [38], orthostatic tolerance [39], balance [40,41], prevention of falls [40], and functionality [41]. Based on our results, one can choose between MJ and SJ when aiming at activating the plantar flexors. It is interesting to note that previous studies showed that MJ exercises, like leg press and squat, are able to promote adaptations in quadriceps, hamstrings, and gluteus [52,53,54]. If lower body MJ exercises prove to be efficient also for plantar flexors, they can provide a time-efficient approach, since their performance can be sufficient to train most lower body muscles. In addition, a significant correlation was verified between exercise conditions for the lateralis gastrocnemius and the soleus, respectively, while no correlation was verified between exercise conditions for the medialis gastrocnemius. To the authors’ knowledge, there are no clear reasons to explain these differences between muscle groups.

We acknowledge that caution is required when extrapolating an acute effect onto a chronic change. Whilst some studies suggest that activation might be important for muscle adaptations [30], others suggest that electromyography cannot be necessarily linked to gains in muscle size and strength [55]. However, it has been suggested that mechanotransduction is likely to occur only in muscle fibers activated during exercise [31]. This suggests that high levels of muscle activation produced from repeated contractions can provide stimulation to the muscle, especially when combined with other factors, such as mechanical and metabolic stress, muscle swelling, etc. [31,32,33,34,35]. Therefore, the present study can provide a rationale for further studies aimed at comparing the long-term effects of MJ and SJ in plantar flexors’ function and morphology.

Based on the results of the present study, the leg press exercise can be recommended to promote muscle activation of the plantar flexors similarly to calf raises. Considering that MJ exercise involves other muscles such as quadriceps, hamstrings, and gluteus, exercise professionals should consider their use as a time efficient approach for exercise prescription.

## Figures and Tables

**Table 1:** Characteristics of the participants.

Variables	Mean ± Standard Deviation
Age (years)	27.1 ± 3.6
Weight (kg)	82.7 ± 6.6
Height (cm)	177.5 ± 5.2
Resistance training experience (years)	3.6 ± 1.4
